# Nanofluidic digital PCR for the quantification of Norovirus for water quality assessment

**DOI:** 10.1371/journal.pone.0179985

**Published:** 2017-07-27

**Authors:** Silvia Monteiro, Ricardo Santos

**Affiliations:** Instituto Superior Técnico, Laboratório Analises, Universidade Lisboa, Lisbon, Portugal; George Mason University, UNITED STATES

## Abstract

Sensitive detection of water- and foodborne enteric viruses is extremely relevant, especially due to the low concentrations in which they are found. Accurate and sensitive detection of Norovirus, the primary responsible for water- and foodborne outbreaks, is of particular importance. Quantification of Norovirus is commonly performed by quantitative RT-PCR (RT-qPCR). In recent years a new platform was developed, digital PCR, that quantifies without the need for a standard curve thus decreasing the errors associated with its utilization. The platform developed by LifeTechnologies, QuantStudio 3D Digital PCR is amongst the least studied digital platform and although it allows the direct detection of DNA targets it requires a two-step RT-PCR for the detection of RNA targets. In this work we developed a new protocol able to detect Norovirus using a one-step digital PCR reaction (RT-dPCR). The performance of the newly developed one-step digital PCR was compared to RT-qPCR for the detection of Norovirus genogroup I and genogroup II. The sensitivity of RT-dPCR was identical to that of RT-qPCR, and the quantitative data determined by both methods were not significantly different for most samples. This one-step absolute quantification approach is a useful tool to minimize the time spent currently using this particular platform to amplify viral RNA and to standardize quantification of enteric viruses in food and environmental samples. This study proved the usefulness of the newly developed RT-dPCR protocol for a sensitive and accurate detection of low-copy targets.

## Introduction

Water is one of the most important resources in the planet and is essential to all life. Increase in population and climate change will increase the demand on existing, but insufficient, freshwater supplies. Therefore, treated wastewater has been used not only for irrigation but also to complement water supplies for non-potable uses in many parts of the world. Nonetheless, the potential reuse of treated wastewater as a source for potable water requires further preoccupation and tighten control with wastewater contaminants, namely pathogenic microorganisms, due to the health risks they may pose.

Currently, there is no agreement regarding the most appropriate standards to regulate water reuse [1]. The California Department of Public Health [2] published the most restrictive regulation, applied to the indirect reuse of wastewater as source of raw drinking water through recharge of groundwater, which requires a 12-log reduction in the concentration of enteric viruses and a 10-log reduction for *Giardia* cysts and *Cryptosporidium* oocysts. Additionally, discharged treated or untreated wastewater can impact negatively irrigation, shellfish-growing, and recreational waters [3]. Therefore, wastewater treatment can be considered a significant control point to limit the extent of microbial contamination of marine environment [4].

Water- and foodborne enteric viruses, particularly Noroviruses (NoV), represent a major risk to public health. NoV are the main responsible for the majority of non-bacterial gastrointestinal illnesses worldwide [5]. They are transmitted mainly by the oral-faecal route either by the ingestion of contaminated food and water or by person-to-person contact. Given the low concentrations at which these viruses are found in the environment, an accurate determination of the efficacy of wastewater treatment processes is paramount.

The ‘gold standard’ method for the determination of viruses infectivity is still cell culture. However, there is currently a lack of reliable cell culture system for the detection of human NoV. Additionally, the low contamination levels of food and water matrices impairs the use of cell culture. Traditionally, real-time RT-PCR has been one of the most powerful tool due to elevated sensitivity, specificity and speed. Quantitative RT-PCR (RT-qPCR) has been therefore the method of excellency for viral load quantification. Quantification is based on a standard curve that requires careful calibration and consistent source material. This has serious drawbacks creating inter-laboratory variability due to differences in the used standard materials, standard curve creation and potential analysis subjectivity [6]. Digital PCR (dPCR) is a new approach to nucleic acid detection and quantification offering an alternate methodology to RT-qPCR. Digital PCR works by partitioning a unique sample into thousands of individual reactions running in parallel. Following amplification the total number of target molecules are calculated, through Poisson statistics, with no need for external reference standards [7–9]. Furthermore, this platform may decrease the levels of inhibitors linked to matrix components present in food and environmental samples [10]. The digital platform consists either of a micro/nanofluidic-based or a droplet-based approach. The most studied platforms are the microfluidic-based Biomark^TM^ HD system (Fluidigm) and the droplet-based QX100^TM^ and QX 200^TM^ Droplet Digital PCR (Bio-Rad). QuantStudio 3D Digital PCR system, developed by Life Technologies, has been used to a less extent specially for viral load quantification. The detection of RNA viruses by this particular platform is rather time-consuming since it requires the production of cDNA before dPCR amplification. So far, it was impossible to perform a one-step digital RT-PCR (RT-dPCR) reaction using this platform since no kit has been commercialized by the manufacturer and no protocol was developed for this use. The use of a two-step procedure not only is more time consuming compared to RT-qPCR but also includes further steps that may reduce sensitivity.

In this study, a one-step nanofluidic digital RT-PCR was developed to determine the potential of this new approach for quantification of food and environmental viruses. Reference material for NoVGI and NoVGII was used to test the potential use and sensitivity of a methodology that is not contemplated by the platform manufacturers. The sensitivity and accuracy of the newly designed protocol of RT-dPCR for the detection of NoVGI and NoVGII in raw and treated wastewater was evaluated and compared to RT-qPCR.

## Materials and methods

### Reference materials

cDNA from NoVGI and NoVGII corresponding to the positions 5260–5410 and 4981–5135 positions of the genome sequences M87661 and X86557, respectively, were cloned in CloneSmart blunt cloning kit (Lucigen, US) and replicated in *E*. *cloni* 10G Duo’s (Lucigen, US). Purification of high quality DNA plasmids was performed using the Qiagen Plasmid Midi kit (QIAGEN, Germany). Digestion of plasmids, reverse transcription, removal of DNA impurities and quantification was performed according to previously established protocols [11]. Aliquots of NoVGI and NoVGII standards were kept at -80°C.

### Concentration of virus from raw and treated wastewater

Recovery of viral particles from raw wastewater was conducted as described previously [12]. Briefly, 42 mL of raw wastewater were ultracentrifuged at 110,000 *g* for 1 h at 4°C to pellet all viral particles together with suspended solids. The supernatant was discarded and sediment was resuspended by mixing with 3.5 mL of 0.25 N glycine buffer (pH 9.5) and placed on ice for 30 min, followed by addition of 3.5 mL 2 x PBS and new centrifugation at 12,000 *g* for 20 min. Viral particles were finally pelleted by ultracentrifugation at 110,000 *g* for 1 h at 4°C and resuspended in 5 mL of 1 x PBS.

Viruses were concentrated from treated wastewater using a low cost procedure as described previously, with minor modifications [13]. Briefly, pre-flocculated skimmed milk solution was added to 300-mL of treated wastewater at pH 3.5 to a final concentration of 0.01%. Pre-flocculated skimmed milk solution was prepared by dissolving 1 g of skimmed milk powder in 100 mL of sterile distilled water and pH adjusted to 3.5 with HCl 1 N. Samples were stirred for 8 h at room temperature to enable the attachment of viral particles to skimmed milk flocs. The flocs were pelleted by centrifugation at 7000 *g* for 30 min at 12°C, and the pellet was dissolved in 10 mL of phosphate buffer (pH 7.5) after discarding the supernatant carefully. Samples were stored at -80°C until further analysis.

### Viral RNA extraction

Following concentration, samples were processed using the QIAamp Viral RNA Mini Kit (QIAGEN, Germany) according to the manufacturer’s instructions. Extracted nucleic acids were eluted in 2 x 40 μL of elution buffer and stored at -30°C.

### Primers and probes

The set of primers and probes used to detect NoVGI and NoVGII are those described in IST/TS 15216–1 [14]. All primers and probes were purchased from Eurofins (Germany).

### Real-time PCR conditions

RT-qPCR amplifications were conducted on a 7300 Real-time PCR system (Applied Biosystems, US). Reactions were performed in a 25 μL reaction mixture containing 1 X RT-PCR buffer, 1.67 μL of detection enhancer and 1 μL RT-PCR enzyme mix, elements of the AgPath-ID^TM^ One-step RT-PCR, 800 nM of each primer, 200 nM of probe and 5 μL of RNA template. One-step RT-qPCR program involved a 10 min reverse transcription of RNA at 45°C, followed by a 10 min initial denaturation at 95°C and finally 40 cycles of 15 s at 95°C and 45 sec at 60°C. Positive and negative controls were included with each set of reaction mixtures. Viral standard was diluted and dilutions were tested in triplicate. Quantification dynamic range varied between 2.0E +04 and 2.0E -01 gc/ μL for NoVGI and NoVGII. A standard curve was generated for each target (NoVGI and NoVGII) resulting from serial dilution in nuclease-free water. Raw and treated wastewater samples were tested in duplicate. The slopes (*S*) of the regression lines were used to calculate the amplification efficiency (*E*) of the RT-qPCR reactions, in accordance with the formula *E* = 10^(-1/s)^– 1 to determine the performance of each assay.

### Digital PCR conditions on QuantStudio 3D Digital PCR system

One-step RT-digital PCR (RT-dPCR) amplifications were carried out on QuantSudio 3D Digital PCR System (ThermoScientific, US). Reactions were conducted in a 15 μL reaction mixture containing 1 X RT-PCR buffer, 0.99 μL of detection enhancer and 0.6 μL RT-PCR enzyme mix, elements of the AgPath-ID^TM^ One-step RT-PCR, 800 nM of each primer, 200 nM of probe and 3 μL of RNA template. Positive and negative controls were added in each run. Viral standards were analysed in triplicate and environmental samples run in duplicate. One-step RT-dPCR program was developed to carry out amplification of NoVGI and NoVGII and included a 10 min reverse transcription at 45°C, followed by a 10 min denaturation step at 96°C, 39 cycles of 2 min at 60°C and 30 s at 98°C, and a final elongation step for 2 min at 60°C.

QuantStudio 3D Digital PCR instrument (ThermoScientific, US) was used to count the number of positive wells out of the total number of well per chip and the Applied Biosystems™ QuantStudio™ 3D AnalysisSuite™ Cloud Software were used to analyse and refine the data derived from QuantStudio 3D Digital PCR instrument. Poisson distribution was used to estimate the average number of copies per chip [7, 15].

### Statistical analysis

All statistical analysis were conducted with IBM SPSS Statistics 22 software (IBM, US). RT-qPCR and RT-dPCR data were converted into a logarithmic format and the non detected samples were converted into the limit of detection for each method. The comparison between RT-qPCR and RT-dPCR graphics were obtained by calculation of the mean value and standard deviation for each sample and both values are represented. One-way analysis of variance (ANOVA) was used to compare quantification of NoVGI and NoVGII as determined by RT-qPCR and RT-dPCR in raw and treated wastewater.

## Results

Optical density measurement provided a value of 2.0 E+06 genome copies for both viruses. Digital RT-PCR sensitivity and accurate quantification was evaluated on serial dilutions of RNA of NoVGI and NoVGII (Table 1). Table 1 represents the total number of genome copies as determined by OD measurement for each genogroup, followed by the number of positive replicates as determined by RT-qPCR and the correspondent mean concentration and positive replicates obtained by RT-dPCR. Sensitivity of RT-dPCR was similar to that of RT-qPCR with approximately 2.00E -01 gc/reaction of NoVGII. Sensitivity for NoVGI was improved by approximately 1.0-log by RT-dPCR and all three replicates for the dilution 0.2 gc/uL were positive with the digital PCR against only one positive for real-time RT-PCR. Variation in results obtained by RT-dPCR decreased as the number of copies in the target decreased, with very low variation for theoretical concentrations between 2.00E -01 and 2.00E +01 gc/reaction mixture of NoVGII. The number of genome copies as measured by RT-dPCR were between 0.1 to 0.6 log_10_ lower than that obtained by RT-qPCR, with the exception of the lowest concentration where RNA detected via RT-dPCR was 0.2log_10_ higher than the expected number of copies calculated by RT-qPCR. For NoVGI variance of genome copies between platforms were found to be between 0.09 and 0.92-log_10_. The data showed that the sensitivity of RT-dPCR was either comparable to that of RT-qPCR (NoVGII) or slightly higher (approximately 1 log_10_; NoVGI)). To effectively compare both quantification methods, the reaction chemistry used was identical. Although the manufacturer from the dPCR platform does not provide a one-step reverse transcription solution, the results achieved demonstrated that a commercially available one-step reverse transcription mastermix can be used to detect RNA genome with this platform. The numbers of genome copies measured by RT-dPCR were similar to those measured by RT-qPCR, with the exception of higher genome copy numbers.

**Table 1:** Sensitivity of the RNA of Norovirus genogroups I and II (NoVGI and NoVGII) by RT-qPCR and RT-dPCR.

Copy number of reference material RNA by OD measurement	NoVGI		Copy number of reference material RNA by OD measurement	NoVGII	
	RT-qPCR positive samples	RT-dPCR Mean ± SD (positive samples)		RT-qPCR positive samples	RT-dPCR Mean ± SD (positive samples)
2.00E +04	3/3	2.31E +03 ± 2.03E +02 (3/3)	2.00E +04	3/3	5.43E +03 ± 1.26E +03 (3/3)
2.00E +03	3/3	2.63E +02 ± 1.09E +01 (3/3)	2.00E +03	3/3	9.19E +02 ± 1.01E +02 (3/3)
2.00E +02	3/3	2.52E +01 ± 1.91E +00 (3/3)	2.00E +02	3/3	9.63E +01 ± 1.34E +01 (3/3)
2.00E +01	3/3	3.51E +00 ± 3.90E -01 (3/3)	2.00E +01	3/3	1.18E +01 ± 1.16E +00 (3/3)
2.00E +00	3/3	9.46E -01 ± 1.60E -01 (3/3)	2.00E +00	3/3	1.53E +00 ± 3.40E -01 (3/3)
2.00E -01	1/3	2.52E -01 ± 8.70E -02 (3/3)	2.00E -01	2/3	3.45E -01 ± 1.10E -02 (2/3)

Results are expressed in Log genome copies per μL, calculated by RT-dPCR. Therefore, for each sample, the number of expected genomic copies determined by the manufacturer was compared directly with the number of genomic copies determined by RT-dPCR. Therefore, for each standard, the number of copies determined by OD and analysed by RT-qPCR were directly compared to the copies determined by RT-dPCR. All experiments were conducted in triplicate and the mean of the three replicates was used for the reproducibility.

**Fig 1:**
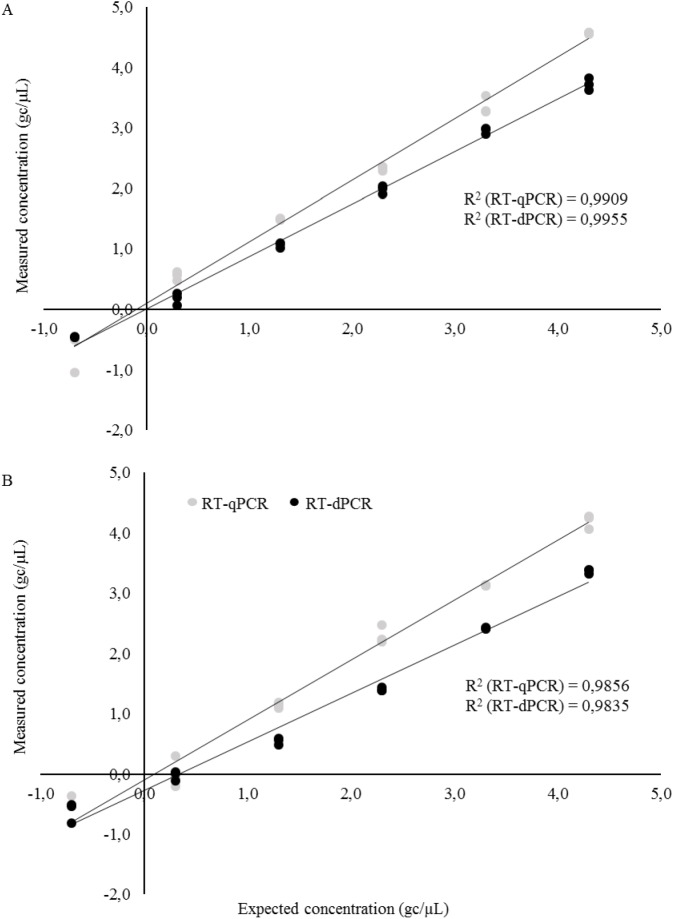
**Regression plots representing linearity for RT-qPCR and RT-dPCR for NoVGI (A) and NoVGII (B) (RT-qPCR: grey circles; RT-dPCR: black circles).** The x-axis indicates expected concentration based on OD measurements for NoVGI and NoVGII as described in Materials and Methods, and y-axis is the measured concentration for both detection methods.

In the dPCR equipment used, the reaction is partitioned into 20,000 nanowells enabling a theoretical dynamic range of approximately five orders of magnitude. The dynamic range of the RT-dPCR assay was determined using decimal dilutions of viral RNA. Linear response was observed for both viruses with the two PCR systems (RT-qPCR vs RT-dPCR) (Fig 1). However, the platform better correlating with RNA genome copies was dependent on the virus. A better correlation with the expected NoVGI genome copies was achieved using RT-qPCR (R^2^ > 0.985) whereas NoVGII genome copies as measured by RT-dPCR showed better correlation with the expected number of copies (R^2^ > 0.996) (Fig 1).

Interestingly, for NoVGI, RT-dPCR showed a superior repeatability to RT-qPCR, as indicated by the coefficient of variation of genome copies between replicates (Fig 1). The repeatability between replicates for NoVGII was similar for both platforms with an excellent repeatability for the lower part of the dynamic range (0.2 gc/ μL) corresponding to a variation of approximately 2% with concentration of 3.45E -01 ± 7.07E -03 gc/ μL.

RT-dPCR and RT-qPCR assays were carried out on environmental samples. Analysis of the qualitative data obtained for NoVGI demonstrated accentuated differences between both methods (Table 2). All samples were positive by RT-dPCR while two out of six raw wastewater (RWW) and two out of six treated wastewater (TWW) were negative by RT-qPCR. On the other hand, RT-qPCR and RT-dPCR produced comparable results for NoVGII (Table 3). Analysis of variance following one-way factor was conducted in order to compare quantification by both methods. The number of genome copies determined by RT-dPCR did not vary significantly from RT-qPCR (*p >* 0.05), except for three RWW samples (RWW2 (*p* < 0.05); RWW4 (*p* < 0.05); RWW5 (*p* < 0.05)). Four samples (two RWW and two TWW samples) presented statistically significant results between the genome copies measured by RT-qPCR and RT-dPCR (RWW5 (*p* < 0.05); RWW6 *p* < 0.05); TWW3 (*p* < 0.05); TWW6 (*p* < 0.05)).

**Table 2:** Quantification of NoVGI in raw and treated wastewater by RT-qPCR and RT-dPCR.

	NoVGI		Quantification NoVGI RT-dPCR vs RT-qPCR
Sample name	RT-qPCR (Log_10_ genome copies)	RT-dPCR (Log_10_ genome copies)	Mean (Log_10_ (NoVGI)_RT-qPCR_−Log_10_ (NoVGI)_RT-dPCR_)
RWW1	< LOD	2.93 ± 0.13	> -1.10
RWW2	2.50 ± 0.07	2.23 ± 0.03	0.27
RWW3	2.49 ± 0.24	2.78 ± 0.02	-0.29
RWW4	3.65 ± 0.03	2.88 ± 0.02	0.77
RWW5	3.29 ± 0.04	2.64 ± 0.08	0.65
RWW6	< LOD	2.63 ± 0.06	> -0.80
TWW1	2.13 ± 0.54	1.94 ± 0.33	0.19
TWW2	< LOD	1.99 ± 0.41	> -0.71
TWW3	2.51 ± 0.21	1.98 ± 0.01	0.53
TWW4	< LOD	1.68 ± 0.01	> -0.40
TWW5	2.21 ± 0.14	2.18 ± 0.02	0.03
TWW6	2.12 ± 0.03	2.04 ± 0.03	0.08

**Table 3:** Quantification of NoVGII in raw and treated wastewater by RT-qPCR and RT-dPCR.

	NoVGII		Quantification NoVGII RT-dPCR vs RT-qPCR
Sample name	RT-qPCR (Log_10_ genome copies)	RT-dPCR (Log_10_ genome copies)	Mean (Log_10_ (NoVGII)_RT-qPCR_−Log_10_ (NoVGII)_RT-dPCR_)
RWW1	3.42 ± 0.17	3.60 ± 0.12	-0.18
RWW2	3.05 ± 0.09	3.03 ± 0.01	0.02
RWW3	4.30 ± 0.02	4.04 ± 0.16	0.26
RWW4	2.84 ± 0.05	2.51 ± 0.12	0.33
RWW5	2.53 ± 0.08	3.44 ± 0.13	-0.91
RWW6	3.61 ± 0.09	3.24 ± 0.02	0.37
TWW1	3.11 ± 0.10	3.22 ± 0.03	-0.11
TWW2	< LOD	< LOD	-
TWW3	2.73 ± 0.07	2.08 ± 0.03	0.65
TWW4	< LOD	1.38 ± 0.03	> -1.10
TWW5	1.83 ± 0.22	1.38 ± 0.00	0.45
TWW6	2.69 ± 0.07	2.33 ± 0.03	0.36

Differences between the two methods were calculated and the results for each sample are displayed in Tables 2 and 3. For NoVGI quantification, the differences varied between 0.27 and > 1.10 in RWW and between 0.03 and > 0.71 for TWW. Moreover, the number of samples with one method providing values superior to the other was similar (seven for RT-qPCR vs five for RT-dPCR). The range of variance for NoVGII in RWW was 0.02 to 0.91 and in TWW was 0.11 to > 1.10. For NoVGII, seven samples presented higher copy number when obtained by RT-qPCR against four by RT-dPCR.

**Fig 2:**
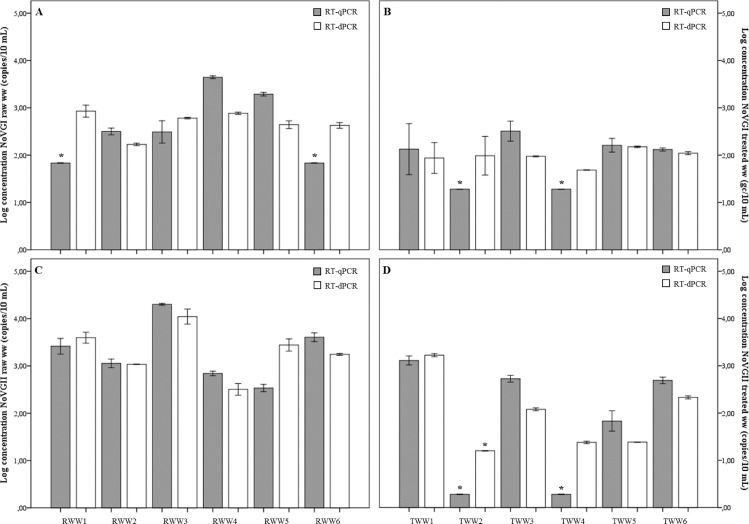
**Variability of RT-qPCR and RT-dPCR quantification for NoVGI RWW (A), NoVGI TWW (B), NoVGII RWW (C) and NoVGII TWW (D).** Values that are below the limit of detection are represented by a *.

A major advantage that RT-dPCR appears to have in comparison to RT-qPCR, apart from absolute quantification, is precision. To compare precision of both detection methods (RT-qPCR vs. RT-dPCR), the coefficient of variation between replicates was evaluated (Tables 2 and 3, Fig 2).

Although norovirus copy number determined by both methods provided comparable results, lower variability was observed for RT-dPCR indicative of a higher precision. For NoVGI genome copies quantification, only one sample (RWW5) showed greater variance between replicates for RT-dPCR. The quantification of NoVGII genome copies by RT-qPCR showed higher precision in only three samples (RWW3, RWW4, and RWW5).

## Discussion

Environmental water quality deterioration is usually due to discharge of wastewater. Treated wastewater may contain pathogenic organisms, such as viruses, that are present in low numbers but may nonetheless pose risks to human health. Additionally, as a result of population growth and climate change the need for extra sources of freshwater is increasing and treated wastewater is in consideration. However, extremely sensitive and precise methods are necessary for the detection of low copy number pathogenic viruses to guarantee the complete absence of false negative results and therefore corresponding risks to human health.

In the absence of a robust cell culture system, the current “gold standard” for the detection of Norovirus is RT-qPCR. RT-qPCR is a sensitive and precise technique capable of giving results in a couple of hours. Nevertheless, there is a growing interest in the application of a newly designed digital platform for the quantification of enteric viruses in water and food matrices. To this end, a microfluidic-based digital PCR (RT-dPCR) was compared to RT-qPCR for detecting and quantifying Norovirus genogroup I and genogroup II in raw and treated wastewater.

The quantification of NoVGI and NoVGII standards by RT-dPCR was of equivalent or higher sensitivity to RT-qPCR. Data from standard curve indicated that for higher genome copy numbers RT-qPCR provided higher results, which is in agreement with previously published studies [10; 11]. Nonetheless, in environmental water samples and food matrices, viruses are traditionally less abundant than these concentrations. The opposite, however, occurred when considering lower concentrations, an observation in line with previously published data [10]. One potential cause for the small disparity between quantification by RT-dPCR and RT-qPCR, particularly for the highest concentration, could be the theoretical dynamic range of the digital platform that is limited by the number of well in the chip (20,000) indicating that the number of NoV genome copies for this concentration was above the platform upper limit of quantification, creating oversaturation of the chip. Additionally, RT-qPCR provides a relative quantification based on a standard curve. The absence of a cell culture system for the quantification of Norovirus implies that the quantification of a standard is performed indirectly through UV spectrophotometry or comparison to a previously established standard curve. This may lead to errors with possibility for an overestimation in the quantification of the standards [16–18] since the analytical precision can be problematic and several assumptions, including the real number of gene copies per cell and conversions to copy numbers, are made [19–20]. The selection of method for quantification of the standard material, qPCR can yield an estimated increase or decrease of 50% in the target copy numbers [20–22]. To properly conduct the comparison between platforms, reaction mixture was similar but the volume of sample used for RT-dPCR was lower than for RT-qPCR. Although a smaller volume was tested a similar or even greater sensitivity was achieved in agreement with previous publications [23–24]. The digital platform produced more accurate measurements not only for standards but also for wastewater samples. Similar results have been previously demonstrated for RNA and DNA targets [10, 18, 20, 24–26]. A previous study has shown that for the lower part of the dynamic range, RT-ddPCR repeatability was unmatched by RT-qPCR with a coefficient of variation below 15% for genome copy concentrations ranging from 54.4 and 6 rotavirus/10 μL reaction [10], corroborating the results described in this study.

Absolute quantification of NoVGI and GII by RT-dPCR showed quantitative agreement to RT-qPCR although the latter provided generally slightly higher virus concentration than did RT-dPCR. This may be indicative of a possible overestimation of Norovirus concentration measured by RT-qPCR, a result described previously [18,24]. For routine analysis of Norovirus and particularly of Norovirus genogroup I, RT-dPCR has been shown to be the most cost-effective and sensitive platform, which is in agreement with previous publications [24, 27]. On the other hand, qualitative analysis showed a higher degree of false-negatives followed detection by RT-qPCR. This is probably due to the higher accuracy and sensitivity of the digital PCR platform since it does not rely on amplification efficiency, which can be affected by several conditions. Distinct PCR components are known to play an important role on the efficiency of qPCR, which may cause substantial differences in the Cq values [28]). In addition, a higher tolerance against PCR inhibitors was already reported [29]. Inhibition in a RT-qPCR occurs mainly through DNA/RNA unavailability or reduced PCR efficiency [30], which causes an increase in the Cq conducting to an underestimation or even a non detection by RT-qPCR. dPCR is an endpoint reaction where a decreased amplification efficiency can still be detected providing a positive result. Additionally, partitioning the sample into thousands of independent and parallel nano-reactions reduces the impact of inhibitors [10–11, 31–33]. Amplification efficiency may also be affected by sequence mismatches in the targeted regions for primers and probes [27]. A more robust technique, such as RT-dPCR, can help to diminish the issues of qPCR inhibitions envisaging its use of quantification of low target concentrations in environmental waters since inhibitions is one of the major drawbacks for validating molecular tools in food and environmental water applications [34]. A key advantage is that RT-dPCR performance makes it appropriate for the quantification of low genome copy numbers expected in water and food matrices. The direct quantification without the use of standard curves may lower the costs per sample. The infrequent usage of RT-qPCR increases the costs simply through the requirement of a standard curve to determine quantification. The higher precision exhibited by RT-dPCR may also diminish costs as the number of replicates is an important element for low concentration or non-detect data interpretation as measured by qPCR [35]. The advantages of RT-dPCR could be attractive to food and water quality monitoring agencies that usually have few economical resources.

However, dPCR may not overcome the issue with nucleic acid availability or even highly inhibitory samples and the molecular drop-out where the target is not amplified is a possible issue [33, 36]. Moreover, RT-dPCR has a higher limit cut-off, a situation not encountered for RT-qPCR, which indicates that dilutions are required to guarantee that the number of genome copies falls within the optimal range [37] increasing an extra variability in the detection. Another important scenario is related to the partitioning of targets in the chip nanowells. Targets with multiple gene copies per genome can be underestimated by dPCR if the copies are not separated previous to dPCR reaction.

In conclusion, this study demonstrated that a commercially available one-step RT-PCR kit could be used in the digital platform with similar or improved results compared to RT-qPCR and confirms the encouraging usefulness of RT-dPCR to address water quality. Although the promising good results, further work is required, which will include field case studies and application in food, to fully evaluate the complete range of benefits and limitations.
